# Engaging English- and Spanish-speaking older adults with and without possible cognitive impairment in advance care planning group visits: Protocol for the ENgaging in Advance Care Planning Talks (ENACT) Randomized Controlled Trial

**DOI:** 10.21203/rs.3.rs-6560971/v1

**Published:** 2025-06-20

**Authors:** Hillary Lum, Samantha Farro, Elizabeth Juarez-Colunga, Chandni Patel, Brianne Bettcher, Christine Haynes, Amy Huebschmann, David Nowels, Caroline Tietbohl, Jiayuan Shi, Carly Ritger, Prajakta Shanbhag, Kathleen Resman, Rebecca Sudore

**Affiliations:** University of Colorado Anschutz Medical Campus School of Medicine; University of Colorado School of Medicine: University of Colorado Anschutz Medical Campus School of Medicine; University of Colorado School of Medicine: University of Colorado Anschutz Medical Campus School of Medicine; University of Colorado School of Medicine: University of Colorado Anschutz Medical Campus School of Medicine; University of Colorado School of Medicine: University of Colorado Anschutz Medical Campus School of Medicine; University of Colorado School of Medicine: University of Colorado Anschutz Medical Campus School of Medicine; University of Colorado School of Medicine: University of Colorado Anschutz Medical Campus School of Medicine; University of Colorado School of Medicine: University of Colorado Anschutz Medical Campus School of Medicine; University of Colorado School of Medicine: University of Colorado Anschutz Medical Campus School of Medicine; University of Colorado School of Medicine: University of Colorado Anschutz Medical Campus School of Medicine; University of Colorado School of Medicine: University of Colorado Anschutz Medical Campus School of Medicine; University of Colorado School of Medicine: University of Colorado Anschutz Medical Campus School of Medicine; University of Colorado School of Medicine: University of Colorado Anschutz Medical Campus School of Medicine; University of California San Francisco Division of Geriatrics

**Keywords:** Advance care planning, group medical visit, primary care, older adults, cognitive impairment, dementia, randomized controlled trial, mixed methods, implementation

## Abstract

**Background:**

Advance care planning (ACP) helps patients identify and communicate their preferences for medical care and prepares them for decision-making related to future care. While ACP is recommended for older adults with cognitive impairment, few interventions have been tested in primary care for this population. The ENACT trial tests the efficacy of the ENgaging in Advance Care planning Talks (ENACT) Group Visits intervention, which engages older adults with and without possible cognitive impairment in group medical visits focused on the ACP process.

**Methods:**

This two-arm randomized trial compares the efficacy of the ENACT Group Visits intervention arm to a control arm at 6-month follow-up. The trial will enroll at least 480 patients across 8 primary care clinics. Notable inclusion criteria include English or Spanish-speaking, age 70 or older, and no ACP documents in the electronic health record (EHR) in the past 2 years. The Montreal Cognitive Assessment screener will be administered at study enrollment to identify whether the participant has possible cognitive impairment. Participants will be randomized 1:1 to the ENACT Group Visits arm or control arm. The ENACT Group Visits intervention entails two group visits, facilitated by multidisciplinary clinic staff. The control arm entails sending ACP materials via mail. The primary outcome is new documentation of ACP in the EHR at 6-month follow up, and it will be analyzed using logistic regression with random effects for site. Secondary outcomes include patient-level ACP readiness, decision self-efficacy, and quality of communication. The impact of possible cognitive impairment on ENACT intervention efficacy will be examined. Survival analyses will be used to examine time-to-new ACP documentation. Mixed methods, including multiple qualitative methods, will be used to assess acceptability, feasibility, intervention fidelity, and other implementation outcomes of the ENACT intervention among patients with possible cognitive impairment.

**Discussion:**

This study will determine the efficacy of the ENACT Group Visits intervention among diverse older adults, including those with possible cognitive impairment, as evidenced by increased documentation of ACP in the medical record. If efficacious, primary care clinics may implement this ACP intervention that leverages peer interactions and goal setting to support a person-centered ACP process.

**Trial registration:**

Clinicaltrials.gov: NCT05421728. Registered on 13 June 2022, https://clinicaltrials.gov/study/NCT05421728.

## Introduction

### Background and rationale {6a}

Advance care planning (ACP) is “a process that supports adults at any age or stage of health in understanding and sharing their personal values, life goals, and preferences regarding future medical care” (1,2). ACP demonstrates benefits for patients through increased autonomy and peace, for families through less intense grieving and reduced likelihood of developing psychiatric conditions, and for healthcare systems through decreased resource utilization and costs (3-5). ACP is also a critical type of decision-making for individuals with cognitive impairment, especially those with possible or diagnosed Alzheimer’s disease and related dementia (ADRD). Rigorous research exists for evidence-based ACP interventions that target individuals with advanced dementia (6). However, these studies often focus on preparing surrogate decision makers, enrolling individuals living in care facilities who have more advanced dementia, and/or individuals recruited from specialty memory clinics (7-9). There is a relative paucity of ACP studies for individuals with cognitive impairment in primary care settings because most studies specifically exclude individuals with established cognitive impairment, such as a diagnosis of ADRD. Studying ACP in primary care settings offers an individual who may have early cognitive impairment the opportunity to participate in decision-making about future medical care prior to loss of capacity (10,11).

A systematic review shows support for ACP interventions, particularly those which facilitate clinician-patient communication and promote written directives (5,12). Group medical visits are often used in primary care to bring groups of patients together for medical care (i.e., diabetes care, chronic disease care), education, peer support, and patient engagement (13-15). Advantageous outcomes of group medical visits include improved clinician-patient communication and high-quality education, high levels of patient and clinician satisfaction, and increased patient behavior change and self-efficacy.

#### Rationale for a group-based ACP intervention

The ENgaging in Advance Care planning Talks (ENACT) Group Visits intervention was developed to promote ACP as a health behavior among older adults and to integrate ACP into primary care settings using a group-based format (14-16). Group medical visits provide a practical and scalable model to engage older adults, enabling more time for discussion than individual visits, peer-to-peer learning, and structured follow-up. Group medical visits have been shown to improve patient education, enhance satisfaction, and strengthen communication, especially in chronic disease management contexts (13-15).

Compared to other evidence-based ACP approaches such as paper or electronic resources, trained ACP staff or facilitators, or individual clinic visits, the ENACT Group Visits intervention may be an effective process for integrating ACP discussions and evidence-based resources into primary care to promote ACP actions (19,20), as shown in Table 1.

#### Early evidence for ENACT Group Visits intervention

In pilot studies of prototypes of the ENACT Group Visits intervention, the intervention was feasible and effective, including with small groups of individuals with possible cognitive impairment (16,18). In small studies, the ENACT Group Visits intervention increased multiple ACP actions, in addition to ACP documentation. Participants reported increases in detailed ACP conversations 6-months after the group visits (19% to 41%, p=.02) and participants (93%) chose a medical decision maker more frequently than control participants (73%) at 6-months (p<0.001) (17,18).

Due to demands on primary care settings, it is less practical or sustainable to implement an ACP group visit model that exclusively serves individuals with possible or diagnosed cognitive impairment. Therefore, the ENACT Group Visits intervention incorporates considerations to support inclusion of individuals with both normal cognition and a range of cognitive impairment (i.e., individuals with cognitive difficulties in either a single domain or multiple domain). One strategy is the ability to include a trusted person or care partner in the group visit. Additionally, the intervention utilizes an evidence-based, easy-to-use advance directive and the PREPARE^™^ program because they have been specifically developed to have low cognitive burden and low health literacy requirements (21).

This clinical trial will test the efficacy of the ENACT Group Visits intervention to increase new ACP documents in electronic health records compared to a control group of mailed ACP materials, among older adult patients in primary care settings with or without possible cognitive impairment, which could be due to mild cognitive impairment (MCI) or early ADRD. The study used a validated cognitive screening tool (telephone Montreal Cognitive Assessment [T-MoCA] (24, 27)) during the enrollment process as a proxy measure of global cognitive function. This measure was used to identify possible cognitive impairment in participants; however, the study did not formally diagnose MCI or ADRD based on the screening measure. The study also uses mixed methods to assess acceptability among individuals with possible MCI/ADRD and their care partners. It also aims to identify factors relevant for fidelity and real-world implementation of the ENACT Group Visits intervention in community-based primary care clinics.

### Objectives {7}

The primary goal of the ENACT study is to address research gaps and advance scientific knowledge by integrating a patient-centered ACP intervention into primary care, thereby helping patients to receive medical care that is aligned with their personal values and cultural beliefs. The current trial will include individuals with possible cognitive impairment who have decision-making capacity alongside asymptomatic older adults. Specifically, the trial includes individuals with possible cognitive impairment that may be consistent with MCI or mild dementia, recognizing that many primary care patients have not undergone formal cognitive evaluations or received a cognitive diagnosis.

The trial will test the following research questions:

Main Question #1: Is the ENgaging in Advance Care planning Talks (ENACT) Group Visits intervention effective in increasing new ACP documentation in electronic health records (EHR) as compared to control? The primary hypothesis is that patients in the intervention arm (ENACT Group Visits intervention) will have higher rates of new ACP documentation in the EHR (primary outcome) and will demonstrate increased ACP readiness, decision self-efficacy, and quality of communication (all secondary outcomes) at 6-months compared to control participants.

Main Question #2: Among patients with possible cognitive impairment, does the ENACT Group Visits intervention demonstrate acceptability, feasibility, and effectiveness? The study hypothesis for this question is that individuals who screen positive for possible cognitive impairment and are in the intervention arm (ENACT Group Visits intervention) will have higher rates of new ACP documentation in EHR at 6-months compared to control participants who screen positive for possible cognitive impairment.

The study also aims to identify factors which are important for fidelity and real-world implementation of the ENACT Group Visits intervention in community-based primary care clinics. The study will determine whether and how the ENACT Group Visits are acceptable, feasible, or appropriate for individuals who screen positive for possible cognitive impairment and their care partners. Additional study objectives include identifying clinic characteristics that influence the fidelity, acceptability, appropriateness, feasibility, and possible sustainability of the ENACT Group Visits intervention from the perspective of clinic staff, including primary care clinicians who facilitate the intervention, staff, and leaders.

### Trial design {8}

The ENACT Group Visits intervention integrates multiple theoretical models to guide its structure and goals, including ACP Engagement Theory (patient-level), Collaborative Learning Theory (interpersonal-level), and strengths of group medical visits (clinic-level) (13,23,24). At the patient-level, ACP Engagement Theory, based on Social Cognitive Theory and Behavior Change Theory, defines measurable ACP behaviors such as selecting a decision-maker, articulating values, and discussing care goals with clinicians (2). At the interpersonal-level, Collaborative Learning Theory states that learning occurs through social interaction and is enriched by diverse experiences and knowledge-sharing (24). The purpose of applying these frameworks with ENACT Group Visits intervention is to support ACP actions (including ACP documentation), behavior change processes, and integration into primary care with billable group visits.

This study is a 2-arm multi-site patient-level randomized controlled trial (RCT) to compare the efficacy of the ENACT Group Visits intervention to a control arm of mailed ACP materials at 6-month follow up. Patients will be allocated 1:1 to the intervention or control arms, stratified by presence or absence of possible cognitive impairment based on T-MoCA score, self-reported ethnicity, education level, and site. As an NIH Stage III efficacy study, the trial will be conducted in community-based primary care clinics and involve clinic-based staff as ENACT Group Visits intervention facilitators. We chose a 6-month follow-up period because many participants (in both arms) will have a clinic visit with their primary care provider within this timeframe, providing opportunity for them to engage in ACP. Additionally, our preliminary efficacy study and other ACP interventions have demonstrated efficacy of primary care ACP interventions at 6-months (18,25,26). The study will enrich for the likelihood of participants with possible cognitive impairment by choosing an eligibility criteria of age 70 years or older (given that the prevalence of cognitive impairment due to MCI/ADRD increases with age) and will systematically assess for possible cognitive impairment with the T-MoCA, a well validated cognitive screener (22,27). Note that throughout the manuscript, we refer to participants who are below our specified T-MoCA cut point as having “possible cognitive impairment”; the rationale for this description is that results from a brief cognitive screener are not synonymous with a comprehensive evaluation or cognitive diagnosis. Importantly, this design also aligns with brief cognitive screening that can be done in primary care settings and thus, the study population is more representative of how individuals with possible cognitive impairment can participate in ACP interventions.

## Methods: Participants, interventions, and outcomes

### Study setting {9}

The ENACT study will be conducted in eight community-based primary care internal medicine or family medicine clinics from two health systems, UCHealth and Denver Health, located in the state of Colorado within the United States. These health systems both use the Epic EHR System and have clinics that serve racially and ethnically diverse populations. Participating primary care clinic sites will be chosen through principal investigator (PI) and co-investigator (co-I) outreach to primary care leaders of clinics that serve a significant population of patients who may be eligible (i.e., age 70 years and older) and discussion of their interest in participating in implementing the ENACT Group Visits intervention for their patients. Clinic-level requirements include ability to identify primary care clinicians and staff interested in implementing ACP group visits and availability of a conference room for group medical visits. A full list of study clinics can be obtained at ClinicalTrials.gov NCT05421728.

### Eligibility criteria {10}

The ENACT study includes three types of research participants: patients, care partners of consented patients, and multidisciplinary clinic staff (See Table 2). Briefly, for patients, inclusion criteria include age 70 or older, receiving primary care at a study clinic, no recent ACP documents (within the past 2 years) in the EHR, ability to participate in English or Spanish, and ability to demonstrate study consent. For care partners, inclusion criteria include age 18 or older, preferred language being the same as the participating patient, and being a care partner to a consented patient who screened positive for possible cognitive impairment on the T-MoCA. The rationale, description of use, and interpretation of the T-MoCA is described in section {18a}. Of note, care partners are identified by a consented patient as someone they trust and whom they desire to be involved in the ACP process. Care partners are not required to be biological family members or existing surrogate decision makers. For clinic members, participants could include ENACT Group Visit intervention facilitators, primary care clinicians, clinic staff, clinic leaders, and/or clinic administrators. ENACT Group Visits facilitators at participating primary care clinics can be primary care physicians, advanced practice providers, and/or other clinical staff (i.e., social workers, behavioral health providers, etc.) who are trained to facilitate the ENACT Group Visits intervention.

### Who will take informed consent? {26a}

This section describes how study staff will obtain informed consent from possible patients, care partners, and clinic staff. The informed consent forms are available at ClinicalTrials.gov NCT05421728. Trained study staff will conduct an informed consent process with interested and eligible patient and care partner participants via phone, video using a secure Zoom link, or in-person at primary care clinics. First, study staff will assess eligibility using a brief screening process that asks potential participants about the inclusion and exclusion criteria. Then, study staff will ensure eligible participants have a copy of the informed consent form, and from there will explain the study and proceed with obtaining verbal consent. The study received approval for a waiver of written documentation of consent. The study staff will use a modified, “teach-back” informed consent process (28,29), which was developed for populations with low health literacy levels or cognitive impairment. Adapting principles to improve readability and understandability (30), the informed consent process involves using a consent form written at less than a 6^th^-grade reading level, printing in at least 14-point font, reading the consent form to possible participants verbatim, allowing adequate time for participant questions, and verifying comprehension through repeated and targeted education until comprehension is achieved. Specifically, four comprehension questions will be asked, and if potential participants require more than three attempts to correctly answer the four questions, they will be deemed ineligible due to lack of ability to demonstrate capacity to understand the study. Only after study comprehension is determined, study staff will ask participants for their verbal consent to participate in the study. By design, we will provide all patients the opportunity to demonstrate informed consent because the goal is to include patients who may have cognitive impairment, including MCI/ADRD. Only those individuals who cannot demonstrate the capacity to consent to the study will be excluded. Verbal consent or reasons for declining to participate will be documented.

For care partners, the informed consent process will follow the same structure as for patient participants. If a patient completes the T-MoCA with a score of 17 or less (*Objective Cognitive Assessment of Enrolled Patients,* described in section {18a}), study staff will offer the opportunity to include a care partner in the study (27). Patients will be asked whether they have a care partner, with the question phrased as, *‘we are able to offer participation to a study partner This could be someone who helps you with medical appointments or medical decisions. Is there someone you would want to participate with you?’* If the patient identifies a care partner, study staff will collect the care partner’s contact information, including name, phone number, and e-mail. Once the care partner has been contacted, study staff will first assess eligibility using a brief screening process that asks about each of the inclusion criteria. Then, eligible care partners will be provided with a copy of the informed consent form by mail or e-mail and study staff will explain the study and proceed with a verbal consent process – the same process as described above for patient participants. For clinic members, study staff will email a recruitment invitation to participate in the study with a copy of the consent form. For clinic members who assist with the group visits and agree to participate in a research activity (i.e., periodic reflection, interview, and/or focus group), study staff will review main points from the consent form at the beginning of the research activity and clinic members will provide verbal consent to continue.

### Additional consent provisions for collection and use of participant data and biological specimens {26b}

Patients and care partners who consent to participate in the ENACT Study will be asked if they also agree to be added to a research recruitment database maintained by the study PI that will use data collected in the current study to screen for eligibility of future clinical research studies. Participants who decline will not be added to the recruitment database. No biological specimens will be collected from participants who agree to be added. In accordance with health system privacy and confidentiality policies, any individuals who will observe ENACT Group Visits (i.e., personal acquaintances coming with a patient participant to a group visit) but who are not otherwise eligible to enroll as a care partner will be asked to provide verbal consent for the audiovisual recording of the group sessions. This consent will be specifically for analyzing intervention fidelity.

### Interventions

#### Explanation for the choice of comparators {6b}

To evaluate the efficacy of the ENACT Group Visit intervention, this study will compare the ENACT Group Visit intervention to a control arm where participants will receive evidence-based mailed ACP materials only. This comparator of mailed materials was chosen because the mailed materials are a low cost, population health-based approach that could be feasible for primary care clinics to implement.

### Intervention description {11a}

#### Intervention Arm: ENACT Group Visits intervention

##### Overview

The ENACT Group Visits intervention is a structured, 2-group sessions approach to facilitate ACP among older adults in primary care settings. The intervention has been described in detail, including the human-centered design to adapt the ENACT Group Visits for individuals with MCI (16-18,20). Table 1 above provides an overview of the ENACT Group Visits intervention content for the two sessions (Group A and Group B). The intervention is grounded in ACP Engagement and Collaborative Learning frameworks, as described in the Rationale for a group-based ACP intervention section above. These evidence-based approaches informed the content, facilitation strategies, and overall design of the intervention to support behavior change and promote patient-centered ACP engagement, with changes for the current study described below.

##### Intervention Structure

Patients randomized to ENACT Group Visits intervention will be mailed ACP materials including a cover letter confirming the ENACT Group Visit session dates, the easy-to-read PREPARE^™^ Colorado Advanced Healthcare Directive form, and the PREPARE^™^ instructional pamphlet (31). In addition to receiving mailed information, participants in the intervention arm will receive reminder calls from study staff about the upcoming ENACT Group Visits and to confirm that materials were received. When recruitment occurs at the clinic, the research staff can provide the study materials in person.

Table 3 describes details of the ENACT Group Visits intervention (17), emphasizing the core functions and related structures in the intervention to achieve those functions (32). For any complex intervention, such as the ENACT Group Visits intervention, we specifically describe the core functions as the key components that the intervention aims to achieve. Then, the structures (sometimes referred to as “forms” in implementation science) are the practical processes or ways in which the “functions” are carried out when the intervention is implemented. The core functions of the ENACT Group Visits include peer and expert-based learning, rapport building, ACP education, evidence-based ACP resources, ACP discussions from different healthcare perspectives, individualized ACP goal-setting, ACP documentation, and a reimbursable ACP process.

Structurally, in the ENACT Group Visits, participants will attend two group visits at their primary care clinic, approximately one month apart, each facilitated by a multidisciplinary team comprised of two trained facilitators. The facilitators can be clinic members including physicians, advanced practice providers, social workers, psychologists, peer navigators and/or a study team member (if two facilitators from the clinic are not available). A necessary component is for at least one trained facilitator to bill visits as part of standard clinical outpatient care. Before the group starts, patients will be checked in by clinic staff. During the visits, ENACT Group facilitators will focus on rapport building; fostering an interactive discussion of ACP topics; and ensuring individuals’ values, preferences, and cultural and religious beliefs are welcomed and included. Facilitators will use Collaborative Learning Theory and ACP Engagement Theory to engage participants in an interactive discussion that focuses on core ACP topics and patient-initiated ACP questions and experiences (see Table 3). Each group session will finish with individualized goal setting for ACP actions, including the importance of updating ACP documents in the EHR and communicating preferences with decision makers and health care clinicians. Facilitators will be trained with ENACT Group Visits facilitation guides (Additional file 1) via a training process outlined in section {11c}. Clinical visit documentation will be completed for each participant in the EHR and billed as appropriate (typically an Evaluation and Management (E&M) level 3).

Groups provided in Spanish will be conducted by ENACT group facilitators who are bilingual in Spanish and English languages. All materials have been translated into Spanish using a certified Spanish translator and a back translation method. A bilingual primary care clinician and bilingual/bicultural research assistant will coordinate the cultural adaptation of the ENACT Group Visits intervention, including anticipated modifications to the facilitation guides and/or choice of ACP videos or teaching approaches. Importantly, the evidence-based mailed materials and PREPARE videos are available for Spanish-speaking populations (21).

### Control Arm: Mailed ACP Materials

Patients randomized to control will be mailed ACP materials including a cover letter confirming their participation in the study and encouraging them to discuss ACP with their primary care provider, the easy-to-read PREPARE^™^ Colorado Advanced Healthcare Directive form, and the PREPARE^™^ instructional pamphlet (31). For clinic-based recruitment, study staff can provide the ACP materials in person. For Spanish-speaking participants in the control arm, all materials will be translated into Spanish using a certified Spanish translator and a back translation method.

### Criteria for discontinuing or modifying allocated interventions {11b}

All participants may request to discontinue participation from the study at any time, including patient or care partner participants, regardless of which study arm they have been randomized to in the study.

### Strategies to improve adherence to interventions {11c}

#### Adherence to study procedures:

All research study team members will complete onboarding and ongoing training, as required by the affiliate university, ethics review board, hospital partnerships, and/or sponsor. Study staff who recruit, enroll, and consent patient participants for the study will also be required to complete comprehensive training to ensure fidelity of use of the T-MoCA. The T-MoCA will be assessed during enrollment because the presence or absence of possible cognitive impairment will be used for study allocation/randomization and as a patient-level covariate. The T-MoCA training will be conducted by a licensed neuropsychologist (study co-I) and includes didactics, mock assessment and supervision of administration, and completion of T-MoCA training and certification through www.mocatest.org.

#### Training Procedures and Adherence to ENACT Group Visits intervention:

Several strategies will be used to ensure adherence to ENACT Group Visits intervention protocol. First, all intervention facilitators (at least 2 per clinic) will complete training prior to facilitating the ENACT Group Visits intervention. Training will include orientation to the purposes of the study conducted by the PI or site co-I and study staff, receiving and reviewing the ENACT facilitator guide (Additional file 1), completing the ENACT Group Visits eLearning modules from the University of Colorado (access available upon request at https://cuelearning.org/), and participating in pre-implementation meetings with co-facilitators and study staff. The eLearning modules include six 15-minute online sessions (Overview; Preparing for an ENACT Group Visit; Learning Objectives for Session A; Learning Objectives for Session B; Facilitation Skills; Troubleshooting) that describe the core components and facilitator competencies with embedded video clips from example group visits. Additionally, facilitators will receive a pre-visit reminder email that will include essential materials: facilitator guide, ACP group visit documentation templates to support EHR documentation, patient frequently asked questions (FAQ) document, the ENACT Group Visits intervention communication skills tip sheet, the Colorado PREPARE Advance Healthcare Directive, links to videos to be shown during the visits, patient check-in instructions, and study team arrival details. Before and on the day of group visits, study staff will be available to support facilitators and answer questions. Immediately after an ENACT group visit, facilitators will be asked to participate in a short debriefing session with a study staff member who is also present during the group visit. The debriefing session will be guided by a worksheet with questions that prompt immediate reflections on the intervention. Group facilitators will also be invited to participate in periodic (biannual), virtual 45-minute Learning Collaborative sessions with other ENACT co-facilitators to discuss facilitation tips, questions, and needs. The purpose of the Learning Collaborative is to provide ongoing support for facilitators and enhance fidelity to the ENACT Group Visits intervention.

### Relevant concomitant care permitted or prohibited during the trial {11d}

Because the ENACT study will occur in the context of primary care practice, it is possible that each clinic may initiate or change their ACP practices that are offered concomitantly with the current trial. Also, typical ACP supports by clinic staff will vary by clinic. Examples of common ACP support efforts in the two health systems include: integration of ACP discussions as part of Annual Medicare Wellness visits, distributing MDPOA forms as part of the check-in process, and/or telephone outreach by clinic team members (i.e., social workers) from a population-based clinic list of patients. The current study aims to mitigate bias from usual ACP supports by utilizing a randomized controlled trial design such that both trial arms will be equally impacted.

### Provisions for post-trial care {30}

Patients randomized to the control arm will be offered the opportunity to participate in the ENACT Group Visits at their primary care clinic, following completion of the 6-month follow-up. These ACP group visits will be delivered by the primary care clinic’s trained intervention facilitators but will not be assessed for research-related activities. All participants in the study will be given information for how to contact the study staff and the ethics review board if they have any questions or experience any distress related to study activities. Participants will also be directed to contact their primary care clinic if they have questions related to ACP.

### Outcomes {12}

#### Primary outcome:

The primary outcome of the ENACT trial is a patient-level pragmatic outcome of new ACP documentation (yes/no) in the EHR at 6-months, including advanced directives (i.e., easy-to-read advance directive, medical durable power of attorney forms, living wills) or medical orders (i.e., out-of-hospital orders such as POLST forms or CPR directives). New ACP documentation will be analyzed as change from baseline to 6-months post-enrollment and as time to event (based on date of new ACP documentation added to EHR). While ACP documentation in the EHR is only one aspect of ACP, it is a commonly measured quality metric by health systems and value-based payment programs. ACP documentation is also an ACP action step that patients desire to do.

#### Secondary outcomes:

As a compliment to the pragmatic primary outcome of ACP documentation in the EHR, the study will also include an ACP Composite outcome. This outcome is a binary metric which combines new ACP documentation in the EHR and new clinician documentation of ACP in the EHR (based on chart review, see section {18a}. There are several other secondary outcomes that will be collected in this trial, including from different participant groups (i.e., patients, care partners, and clinic staff members). Patient-level secondary outcomes include readiness for ACP actions, decision self-efficacy, patient report of clinician’s quality of communication, and the ACP composite measure. Readiness for ACP actions will be analyzed using mean score of the four-item ACP Engagement Survey (34). Decision self-efficacy will be analyzed using calculated scores ranging from 0-100 on the Decision Self-Efficacy 4-point Likert Scale (35). Clinicians’ quality of communication will be collected via patient report and analyzed using summary score on the Quality of Communication (QOC) General Communication Skills Scale questionnaire (36,37). All outcomes and additional covariates will be collected as described in Table 4 and section {18a}.

#### Qualitative outcomes:

As part of a mixed methods study, implementation outcomes are feasibility, acceptability, and appropriateness of the ENACT Group Visits intervention among group visit facilitators, clinic leaders, participants with possible cognitive impairment and their care partners (36). Additional process outcomes include intervention fidelity, facilitator and clinic member perspectives of the intervention, adaptations, and sustainment into primary care practices. Detailed data collection methods for the quantitative and qualitative outcomes are described in section {18a}.

### Participant timeline {13}

**Figure 1:**
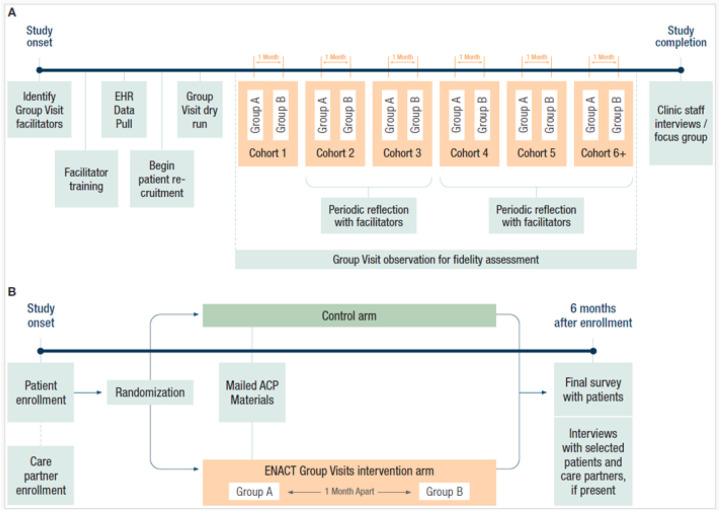
Timeline for clinic (Panel A) and patient and care partner-level study activities (Panel B).

Figure 1A depicts a clinic-level timeline for implementation of the ENACT study, beginning with identification of Group Visit facilitators, continuing with facilitator training, patient and care partner recruitment, conduct of group visits, and end of study assessments. The patient and care partner participants’ timeline is shown in Figure 1B and includes patient and care partner enrollment, randomization to ENACT Group Visits intervention arm or control arm, and 6-month follow up assessments. The data collection process for different types of data is also shown at the clinic and participant levels.

### Sample size {14}

**Figure 2:**
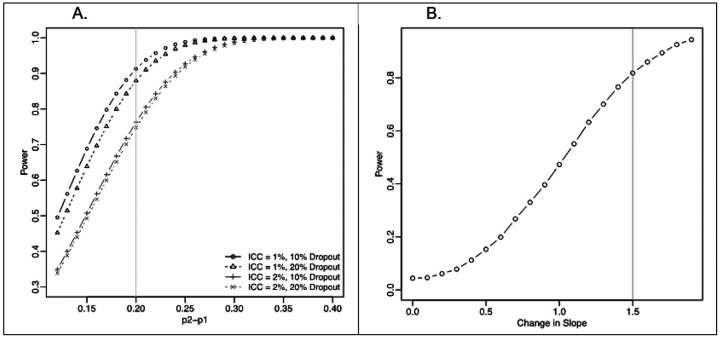
Power estimates of detecting a difference in new ACP documentation. A) Power of detecting a treatment effect in the primary outcome depending on the effect size. B) Power to detect interaction effect between possible cognitive impairment and treatment. ICC = intraclass correlation.

For study question #1 on testing the efficacy of the ENACT Group Visits intervention compared to the control arm, the sample size (n=480; 240 patients per study arm) was chosen to allow detection of at least 20% difference between the ENACT Group Visits arm and control arm in the primary outcome of new ACP documentation (see Figure 2A) at a power of 91% and a 0.05 significance level, while allowing for 10% loss to follow-up, assuming study implementation in 8 primary care clinics. The power calculation adjusts for an intra class correlation (ICC) of 1% to account for possible variability across sites. Although estimates for the effect of ENACT Group Visits intervention on new ACP documentation in primary care do not exist, our preliminary data from the pilot RCT on advance directives suggests an effect size of 26%, so our assumption of a 20% difference is conservative. Even if the ICC is higher at 2%, the power remains at 76% (alpha=.05) with 10% dropout to detect a 20% difference.

Relating to study question #2, the overall sample size allows detection of differential effect sizes by possible cognitive impairment and other possible sources of heterogeneity (such as age, gender). The detectable difference for each age (<75 or ≥75 years), gender level (e.g., female) is in the range of 16-19% (power=80% and alpha=0.05) depending on the proportion of patients in each subgroup (40-60%). If needed, we will use oversampling strategies to ensure at least 15% of the participants have possible cognitive impairment. To calculate power for testing heterogeneity of treatment effect based on possible cognitive impairment, we used simulation (using 2000 datasets) conducted in R86 (43) based on a logistic regression model with an interaction between treatment and possible cognitive impairment (Figure 2B). Based on 15% prevalence (81 patients, assuming a dropout of 10%), the detectable slope is −1.5 with 81% power (0.05). This corresponds to an effect of the intervention (difference) at 6-months of 25% (50% new ACP documentation in control arm vs 75% new ACP documentation in ENACT Group Visits arm) in the non-impaired group and an effect (difference) of 35% in the possible cognitive impairment group (40% new ACP documentation in control arm vs 75% new ACP documentation in ENACT Group Visits arm).

### Recruitment {15}

Recognizing that personal, contextual, cultural and research-related factors affect decisions to participate or stay in a study, and decisions are often influenced by trust, relevance and perceived benefits, the ENACT study uses several strategies to support recruitment. Strategies include involvement of older adult community partners in creating and adjusting recruitment messages, use of consistent branding with plain language and connection to the patient’s primary care clinic, offering flexible hours via phone/video/email and calling from recognized primary care numbers, employing trained Spanish bilingual/bicultural study staff, and iteratively refining study scripts to optimize clarity and person-centeredness of recruitment conversations.

#### Patient recruitment

The ENACT study will primarily use a population-based approach to identify potentially eligible patients in each study clinic. A HIPAA waiver was obtained to allow a clinic-level EHR data report of patients who have at least one primary care clinic visit in the past year, no recent (i.e., from the past two years) ACP document in the EHR, and preferred language of English or Spanish. The report will include potentially eligible patients’ name, language preference, phone number, mailing address, email, and primary care provider name. Then, the study PI and study staff will work with clinic leadership to inform primary care providers (PCPs) about the ENACT study and provide an opportunity to learn about the study’s purpose and patient recruitment process. PCPs will be offered the opportunity to review a list of their patients and the option to “opt-out” any individuals prior to outreach by the study team.

Study staff will mail and/or e-mail eligible patient participants information introducing the study, a copy of the consent form, and instructions on how patients can “opt-out” by phone or email if they do not want to be contacted further. Patients who do not “opt-out” will be contacted up to 3 times by study staff to determine their interest in the study. Study staff will outreach by phone and may also make an in-person approach, within the 3-attempt limit, during a scheduled PCP visit. Study staff will identify upcoming PCP appointments and provide the PCP or Medical Assistant (MA) with an IRB-approved flyer to offer to the patient. Patients may also self-refer into the study (e.g., directly contact study team based on flyers posted in clinics) and will be screened for eligibility using standard procedures. Interested patients will then meet with study staff via phone, video using a secure Zoom link, or in-person at their primary care clinic to complete the consent process (described above in section {26a}) and baseline study activities. For patients indicating that they would like more information about the study, study staff may offer to send the patient a video link that further describes the ENACT Group Visits intervention (English video link: https://www.youtube.com/watch?v=kF_pD8kWKRQ&t=6s; Spanish video link: https://youtu.be/Ez8M2hqd0SE).

#### Care partner recruitment for patients with possible cognitive impairment

Care partners will be invited to participate in the study when a patient has possible cognitive impairment, based on a T-MoCA score of 17 or less. These patients will be directly asked if they have a care partner who can also participate with them in the study. Study staff will then collect contact information of the care partners (e.g., name, email, phone number) from consented patient participants. The consent form will be sent to the care partners, following a phone call from study staff to inquire about interest in participation and eligibility screening. The consent process is described in Section {26a}.

During ENACT Group Visits, patients may occasionally bring a care partner to participate in medical discussions, as they would for any other medical appointment. In these cases, the research team will determine whether the care partner is eligible to enroll in the study (i.e., if the patient has a T-MoCA score of 17 or less). If the care partner is eligible, they will be informed about the study and invited to enroll. If they agree to participate, the same consent process as described in Section {26a} will be followed. In cases where the care partner does not provide verbal consent to participate or is not eligible (t-MoCA score > 17), they will be asked to wait in a separate area to maintain the privacy of the study session.

#### Clinic staff recruitment

Clinic staff involved in group visit implementation will be recruited by emailing information about the study and a copy of the consent form. Up to three attempts will be made to reach the clinic staff by e-mail.

### Assignment of interventions: allocation

#### Sequence generation {16a}

The randomization allocation sequence will be generated by the study biostatistician and analyst for permuted block randomization with random block size using the SAS software (SAS Institute, Cary NC). The randomization sequence will be stratified for patient clinic, presence or absence of possible cognitive impairment based on T-MoCA score, self-reported ethnicity, education level (greater than or equal to 12 years of education), and site. Block randomization ensures the uniform distribution of patient participants in each arm, across clinics, and considering their ethnicity and possible cognitive impairment.

#### Concealment mechanism {16b}

The SAS software-generated allocation sequence will only be known to the study biostatistician and analysts. They will upload the sequence to the REDCap (Research Electronic Data Capture) randomization module (44). The allocation sequence is not accessible to the study staff or PI.

#### Implementation {16c}

For implementation of allocation, patient participants will be enrolled by study staff. Then, REDCap will be used to randomize eligible patient participants using a 1:1 allocation to either study arm. Study staff will then communicate study arm allocation with the enrolled participant, including informing intervention participants of the dates for the upcoming ENACT Group Visit Session A and Session B. Intervention participants will be sent a cover letter that confirms dates of the ENACT Group Visits and includes the mailed ACP materials. They will also be contacted by study staff 2-3 days prior to the group visits as a reminder. Control arm participants will be sent the ACP materials.

### Assignment of interventions: Blinding

#### Who will be blinded {17a}

The current study does not include blinding or unblinding procedures. It is not feasible within a real-world, community-based primary care setting to mask whether a participant is assigned to the ENACT Group Visits intervention or mailed ACP materials.

#### Procedure for unblinding if needed {17b}

Not applicable, this is not a blinded trial.

### Data collection and management

#### Plans for assessment and collection of outcomes {18a}

This section describes plans for assessment of patient, care partner, clinic-level quantitative and qualitative data. Table 4 introduced the key study measures, including primary and secondary outcomes. Spanish-speaking bilingual study staff members will assess patient-level data from patients who prefer to participate in Spanish. Study data will be collected and managed using REDCap electronic data capture tools hosted at University of Colorado (44). REDCap is a secure, web-based software platform designed to support data capture for research studies, providing 1) an intuitive interface for validated data capture; 2) audit trails for tracking data manipulation and export procedures; 3) automated export procedures for seamless data downloads to common statistical packages; and 4) procedures for data integration and interoperability with external sources. The ENACT REDCap data capture tool is available upon request. The qualitative data collection guides are available as described below and in Additional file 2.

##### Patient Data Collection

Patient participants from all clinics will complete baseline assessments after study enrollment. At 6-months after enrollment, patients will be contacted up to three times by telephone to complete final survey questions. At both time points, patient participants will have the option to complete assessments via telephone, secure zoom link, or in-person at their primary care clinic. The option to e-mail the final surveys will also be given to patients who request it.

##### Objective Cognitive Assessment of Enrolled Patients

The Blind/Telephone MoCA (T-MoCA) is a cognitive screening measure that will be administered after study enrollment at baseline to assess for possible cognitive impairment. The T-MoCA was chosen because it is a brief screening tool available in English and Spanish that is well validated, reliable, and used in large scale studies of ADRD (45). The T-MoCA includes all items from the full MoCA, except for the naming, cube drawing, and clock drawing items. The T-MoCA score ranges from 0-22 (24,27) and will be used for stratification and a covariate in this study. We elected to use a cut score of 17, with ≤ 17 reflecting possible cognitive impairment, based on a recently published paper using Youden’s index (which optimizes sensitive and specificity) in diverse populations (30).

##### Patient-reported Quantitative Secondary Outcomes

There are four quantitative secondary outcomes in the ENACT study. The ACP Engagement Survey, collected at baseline and 6-months, is a 4-item survey that assesses ACP readiness for signing papers for a decision maker, talking with a decision maker, talking with the doctor about future care, and signing papers about future care, all on a 5-point Likert scale, and analyzed using mean score (34). The Decision Self-Efficacy Scale, collected at baseline and 6-months, is an 11-item questionnaire that measures self-confidence or belief in one’s abilities in decision making, on a 4-point Likert scale, with calculated scores ranging from 0 to 100 (35). The Quality of Communication General Communication Skills Scale, collected at baseline and 6-months, includes 13 items using a 0-10 scale or a binary yes/no score (36,37). The General Communication subscale will be analyzed using methods adapted for use among older adults with possible cognitive impairment in primary care settings (46).

ACP Composite, assessed at 6-month follow up, is a combined outcome of new ACP documentation in the EHR and clinician documentation in the EHR. Clinician documentation of ACP in the EHR will be measured using a standardized chart review audit process, double coded by two research assistants. Importantly, ACP documentation associated with the ENACT Group Visit Sessions A or B will be excluded, since documentation is expected as part of the ENACT intervention. Two data collectors in each health system will be trained on the chart review protocol, which includes timelines for when to abstract data from Epic into the REDCap database, and definition for variables of interest. We will periodically conduct inter-rater analysis to identify and resolve discrepancies between the two coders and any adjudication of discrepancies will be conducted by a study co-investigator who is a family medicine physician with ACP experience.

##### Patient-reported Covariates

In addition to the objective assessment of cognition using the T-MoCA, subjective cognitive decline will be measured at baseline using 3 binary (yes/no) self-report items, adapted from prior studies (38,39,46): *“Have you noticed changes or problems with your memory or thinking in the last 2 years?”; “Are you concerned about your memory or thinking?”; “Are changes in your memory making everyday life more challenging or difficult?”.* Subjective cognitive decline can be measured with as few as one question (48).

Other covariates include self-reported demographics from patient participants collected at baseline. Demographics includes age, gender, race/ethnicity, marital status, education, care partner status. Preferred spoken language (English, Spanish) will also be collected at baseline. The PROMIS Global Health questionnaire, collected at baseline and 6-months, is a self-rated 10-item scale, which uses a 5-point Likert scale (Excellent to Poor) and a 0-10 scale to assess pain (40).

Prior ACP experience will be collected at baseline using 5 self-report items with a binary (yes/no) scale (26,31): *“Have you made out a will saying how you want your property divided?”; “Have you made funeral arrangements; such as pre-paying for a funeral, buying a burial plot, or making arrangements for cremation?”; “Have you ever filled out an advance directive, living will, or durable power of attorney for healthcare? (If needed: These forms let you write down your wishes for medical care. Some people do not know if they filled them out and this is OK.)”; “Have you ever had to make life threatening medical decisions for yourself; such as, whether to have major surgery (like open heart surgery); to be put on a breathing machine; or to go to the intensive care unit (ICU) of the hospital?”; “Have you ever had to make life threatening medical decisions for someone else; such as, whether they should have major surgery (like open heart surgery); be put on a breathing machine, or to go to the ICU because they were too sick to make their own decisions?”.* Past ACP communication will be collected at baseline using 3 self-report binary (yes/no) questions: *“If you become too sick to make your own decisions, can you think of ANYONE in your life right now, such as family or friends, who MAY be able to help make medical decisions for you?”; “Do you have close friends or family that may have opinions about your medical care if you were to become sick?”;* and *“Have you had discussions with a doctor about treatments you would want if you are too sick to speak for yourself?*”.

Self-report of social determinants of health will be assessed using two items from the PRAPARE screening tool: “*In the past year, have you or any family members you live with been unable to get any of the following when it was really needed?”* with choice options of: food, utilities, clothing, child care, medicine or any health care (medical/dental/mental health/vision), phone, other, none of the above, I choose not to answer this questions; and “*Has lack of transportation kept you from medical appointments, meetings, work or from getting things needed for daily living?”* with choice options of: “no, yes it has kept me from medical appointments, yes it has kept me from non-medical meetings/appointments/work/or from getting things that I need, I choose not to answer this question”(42). The ENACT study used an adapted version of the Medical Outcome Study Social Support survey (11-items) to assess self-report of social factors that will be collected at baseline using a 5-point Likert scale (none of the time to all of the time) (49,50).

##### Patient-level Electronic Health Record Data

At the end of the follow up period for all participants, EHR data will be abstracted from Health Data Compass (UCHealth data warehouse) and from Epic Clarity (Denver Health data warehouse), including primary care clinic site, patient medical record number, patient name, date of birth, PCP, language preference, race, ethnicity, gender, zip code. Zip code will be collected and used to determine the social vulnerability index. Medical conditions (coded in past year vs. Problem list) will be collected at baseline to calculate the Charlson Comorbidity Index (51). Additional factors associated with elevated risk of cognitive impairment will also be assessed, including Body Mass Index (obesity measure), hyperlipidemia, and relevant co-morbid diagnoses (such as diabetes, hypertension, depression, anxiety, and dementia).

##### Patient-level Qualitative Data

At 6-months post-enrollment, a portion of patient participants will be invited to participate in an optional interview asking about their ACP engagement, including prior ACP experiences, personal goals around ACP, benefits and disadvantages of the study and how it impacted the ACP process, and feedback about group visits’ and mailed materials’ content, delivery, and appropriateness for patients with memory or thinking problems. The qualitative team will guide recruitment with a goal of interviewing 7-8 patients with possible MCI per clinic (approximately 2-3 intervention participants and 2-3 control participants). A Spanish bilingual study staff member will conduct the interviews in Spanish, if preferred by the participant. The English interview guide is available in Additional file 2; the Spanish guide is available upon request.

##### Care Partner Data Collection

At baseline, self-reported demographics will be collected from care partner participants, including date of birth, gender, race/ethnicity, language preference, education level, marital status, and zip code. At 6-months post-enrollment, interviews will be conducted with 1-3 care partners of patient participants with possible MCI. The goal will be to purposively sample across all clinics. The interviews will focus on acceptability and appropriateness of the study (ENACT Group Visit intervention arm or mailed ACP materials control arm) from a care partner perspective. Interviews will include questions about prior ACP experiences; care partners’ goals around ACP for the patient; value, benefits, and disadvantages of the study; feedback about group visits’ and mailed materials’ content and delivery; and resources for care partners regarding ACP. A Spanish bilingual study staff member will conduct the interviews in Spanish, if preferred by the participant.

##### Clinic-level Data Collection: Quantitative and Qualitative Data

###### Group Visit Fidelity:

Study staff will conduct fidelity assessments of each group visit to assess the extent to which the delivery of the ENACT Group Visits intervention adheres to the model originally developed and described in Table 3, including its core functions and structures (52). Using video and audio recordings of the group visits, the research team will observe all group visit attendees, including patients, care partners, and facilitators to rank and provide rationale on fidelity to intervention core components. By looking across individual group visit fidelity assessments, qualitative research team members will use rapid qualitative analysis to create a fidelity summary for each clinic and distribute to facilitators of the group visits at the end of group visit implementation at each clinic. Fidelity assessments of group visits conducted in Spanish will be undertaken by trained bilingual study staff, completing data collection in English so that it can be combined and/or compared with other intervention groups.

###### Group Visit Facilitators - Periodic Reflections:

Clinic facilitators will be asked to participate in periodic reflections, which provide “detailed, near-real-time information on projects’ dynamic implementation context,” including events or changes at the clinic, adaptations to the group visit intervention, and team learnings through implementation (53). Facilitators will be provided with an opportunity to reflect on the group visits they have facilitated and problem-solve any challenges. Periodic reflections will be conducted approximately twice throughout each clinic’s study implementation period. Transcripts of audio-recorded periodic reflections will be assessed using rapid qualitative analysis.

###### Clinic Staff - Qualitative and Quantitative Data Collection:

To identify each clinic’s characteristics that influence the ENACT Group Visits fidelity, acceptability, appropriateness, and feasibility of the intervention, qualitative data will be collected from clinic staff including involved clinic leaders, administrators, and front-line staff at the end of each clinic’s study implementation period. Qualitative, individual, semi-structured interviews will be conducted via Zoom or in person with 1-2 clinic leaders and administrators (e.g., medical directors, practice administrators) within three months of finishing patient enrollment into the study at each respective clinic. Focus groups will be conducted via Zoom or in-person with clinic staff involved in group visit implementation (e.g., physician/advanced practice provider facilitators, social worker facilitators, clinic visit schedulers, MAs) within three months of finishing patient enrollment into the study at each respective clinic. Clinic staff will be asked to complete the Acceptability of Intervention Measure, Intervention Appropriateness Measure, and Feasibility of Intervention Measure (36), which are all comprised of 4-items each and use a 5-point Likert scale from Completely Disagree to Completely Agree. These implementation outcomes will be described as mean scores for each scale.

###### Clinic Staff – Sustainment Interviews:

To understand whether and how clinics may choose to sustain group visits after the study concludes, individual semi-structured interviews will be conducted via Zoom or in person with 1-2 staff per clinic either 6-months after the last clinic interviews and focus groups or 8-12 months after the last group visit conducted at the clinic. These interviews will be conducted with key clinic staff who were involved in the decision-making processes around maintenance of group visits or actively help sustain group visits at each clinic. The interviews will elicit information about decision-making around group visit sustainment, descriptions of current group visits, adaptations made to group visits since the study ended, and barriers and facilitators to sustaining group visits at a given clinic. Table 5 summarizes the qualitative data collection across the ENACT study and involving multiple participants, such as patients, care partners, group visit facilitators and clinic staff.

#### Plans to promote participant retention and complete follow-up {18b}

Multiple strategies will be used to promote patient retention for complete follow-up. At the time of enrollment, study staff will request multiple contact numbers and an alternative contact. Participants will be sent ENACT study thank you cards 3 months after enrolling into the study, including a reminder for the 6-month call. Patients will be offered a $25 gift card after completing participation at each time point (baseline and 6-months). To improve retention at the 6-month follow up, if study staff are unable to reach a patient participant within 3 attempts and the individual has an e-mail address on file, the final (6-month) surveys may be e-mailed to them as the fourth and final attempt to complete study activities. If recommended by the clinic team, study staff may seek to meet patients at expected primary care appointments to facilitate completion of the 6-month follow up. Care partner participants who participate in 6-month interviews will also be offered a $25 gift card. Clinic staff participants who participate in periodic reflections will be offered a $50 gift card per reflection (maximum 2 reflections per site). Clinic staff who participate in the focus groups and clinic leaders who participate in the interviews will be offered a $50 gift card.

#### Data management {19}

The study incorporates multiple procedures to ensure accurate and high-quality data management. Data entry will be completed by study staff, all of whom will have completed training and must be approved by the IRB.

##### Data Storage, Security, and Quality

All written and audio/video-recorded data will be stored in locked cabinets and on password-protected, encrypted computers. All consent documentation and quantitative or qualitative study data will be saved in REDCap or the University of Colorado-Denver secured servers. REDCap is a secure web application designed to support data capture for research studies, providing user-friendly web-based case report forms, real-time data entry validation (e.g., for data types and range checks), audit trails, and a de-identified data export mechanism to common statistical packages. The database is hosted at the University of Colorado–Denver Development and Informatics Service Center (DISC), which will be used as a central location for data processing and management. Qualitative data will be collected via University of Colorado-compliant Zoom videoconferencing software or phone channels, or in person in a confidential location convenient for participants.

Data quality will be ensured using a team-based approach. Study staff will conduct data checks, both univariate (range checks), as well as across variables (especially dates) at the time of data entry, including checking for duplicate and missing records. Further, periodical ENACT trial summary reports will be generated by the analyst(s) summarizing accrual and data collection. During the statistical analysis, data quality will be further ensured by assessing completeness (missing values), identifying outliers, verifying consistency across datasets, checking for duplicates, evaluating data types and formats, analyzing data distributions, and using visual exploration techniques to identify possible issues and anomalies within the data.

To ensure qualitative data quality, data collections will be audio-recorded and professionally transcribed by a HIPAA-compliant transcription and translation company (Landmark Associates, Inc.). Spanish language data collections will be translated and transcribed by Landmark, with 20% of all translated transcripts to undergo validation by a bilingual Spanish-speaking study team member. To ensure rigor in qualitative data analysis, a qualitative methodologist and analyst will meet regularly throughout initial coding and matrix populating to refine codes, compare application of codes, refine matrix domains, compare matrix populating from transcripts, and double code 20% of coded transcripts.

#### Confidentiality {27}

Several strategies will be used to manage and protect the privacy of participants and confidentiality of research data. Participants will be assured that their study answers and data will be de-identified. Specifically, any identifying information will be coded, separated from the data, and kept on a password-protected, encrypted server. ACP documents that the patient may complete or bring in during the study will be placed in the patient’s EHR, upon their request. For audio-recorded data, study personnel will redact any spoken identifiers (names, etc.) from transcripts and will label audio-files with a sequential de-identified number.

#### Plans for collection, laboratory evaluation and storage of biological specimens for genetic or molecular analysis in this trial/future use {33}

Not applicable, no samples collected.

### Statistical methods

#### Statistical methods for primary and secondary outcomes {20a}

Intent-to-treat analysis will be the primary analytic approach, including all participants as randomized. Per-protocol analysis will be conducted as a sensitivity analysis, and participants who deviated from the protocol or withdrew from the study will not be included in the analysis. In the per-protocol analysis, data from participants who adhered to the study protocol will be used to compare the intervention and control group outcomes. This will enable assessment of the efficacy of the ENACT Group Visit intervention in condition where participant adherence is high.

Descriptive statistics and tests for differences will be computed for patient characteristics between the intervention arms. For continuous variables, mean, median, standard deviation, range, and percentiles will be summarized. For categorical variables, frequencies and percentages will be reported. T-tests will be used to compare continuous measures and Chi-squared tests will be used to compare proportions of categorical measures between the control and intervention participants.

To evaluate the main study question #1, we will use logistic regression with random intercepts for site to assess the likelihood of having new ACP documentation in the EHR at 6-months. The dependent variable will be the presence or absence of new ACP documentation in the EHR at 6-months, with the ENACT Group Visits intervention as the primary predictor. Additional covariates may be included to adjust for possible confounders. Additionally, we will conduct a time-to-completion analysis based on length of time from date of enrollment to first detection of new ACP documentation, using survival analysis methods including log rank test and Cox regression with frailty terms for site. The analyses will be applied first on the EHR data pull, then be confirmed by the chart-review based data.

We will assess for clinical site differences by comparing demographic variables across sites using ANOVA (for continuous variables) and Chi-squared tests (for categorical variables) as appropriate. We will employ generalized linear mixed models (GLMMs) to incorporate data structures that are longitudinal. Hypothesis tests will be conducted using two-sided tests with a significance threshold of ɑ =0.05. P-values will be reported for all tests. Goodness of fit statistics and model fitting diagnostics will be used to assess for influential points, outliers, and heteroscedasticity.

#### Interim analyses {21b}

Not applicable to the current trial.

#### Methods for additional analyses (e.g. subgroup analyses) {20b}

For evaluation of the main study question #2, we will use a logistic regression model with an interaction term between treatment arm and possible cognitive impairment status, treated as a binary variable, to assess whether the intervention effect differs by cognitive impairment status. The dependent variable will be the presence or absence of new ACP documentation in the EHR at 6-months, with the ENACT Group Visits intervention as another predictor. Additional covariates may be included to adjust for possible confounders.

We will also use qualitative methods to evaluate main study question #2. To assess the acceptability and feasibility of ENACT Group Visits for patients with possible cognitive impairment and their care partners, we will use content analysis to elucidate participants’ perspectives and experiences regarding the intervention arm and the control arm. We will also use rapid qualitative analysis to understand clinic staff perspectives of clinic characteristics (e.g., health system type, preferred language of patient population) related to the implementation outcomes of acceptability, appropriateness, and feasibility, and their perspectives on sustaining group visits at their respective clinics. Finally, we will use rapid qualitative analysis to describe group visit intervention fidelity, including engagement among patients with possible MCI.

To determine key factors that impacted the intervention across multiple clinics, we will use a convergent mixed methods approach. We will use rapid qualitative analysis of periodic reflections, video-recorded group visits, clinic leader interviews, clinic staff focus groups, and sustainment interviews to identify important factors that were discussed by various participants throughout implementation (e.g., clinic structure, staffing, resources, perceived value of ACP group visits). These findings will be integrated with quantitative outcomes (e.g., ACP documentation) to determine patterns in intervention impact.

#### Methods in analysis to handle protocol non-adherence and any statistical methods to handle missing data {20c}

Prior to beginning statistical analyses, we will examine the data carefully to determine whether patterns of missing data seem ignorable (MCAR or MAR) or non-ignorable (MNAR), and we will address these patterns appropriately.

#### Plans to give access to the full protocol, participant level-data and statistical code {31c}

The final protocol at the time of completion of study recruitment, participant-level de-identified dataset, and statistical code will be posted to Open Science Framework (https://osf.io/) and will be made available upon request.

### Oversight and monitoring

#### Composition of the coordinating center and trial steering committee {5d}

##### Composition

Day-to-day support for the ENACT trial is provided by the University of Colorado coordinating team which is comprised of: the study PI, study project manager, study research assistants, study biostatistician and data analysis team, qualitative methodologist and qualitative analysis team, as well as study co-investigators with expertise in ACP, implementation science, and neuropsychology. The ENACT study team meets weekly to address procedural, logistical, and methodological matters for the study. The Colorado Institutional Review Board (COMIRB) is also available to provide day-to-day support and consultation as the study’s ethics review board.

##### Roles and Responsibilities

The PI, with the support of the study team, is responsible for ensuring continuous participant safety during study participation and for identifying and immediately reporting all adverse events and serious adverse events (AE/SAE). The PI is also responsible for monitoring and final decision-making across all aspects of the study. Study monitoring includes focusing on recruitment, baseline comparability of treatment groups (randomization), protocol adherence (including intervention fidelity), completeness of data (including follow-up rates), accrual of primary and secondary outcome data, safety (monitoring, reporting of AE/SAEs), and leading or coordinating all data analysis, interpretation and dissemination. The PI will discuss monitoring in monthly scientific team meetings.

The study project manager is responsible for overall study project management including study team onboarding, training, management, and oversight; fidelity of implementation procedures; safety monitoring and reporting to the PI, and recruitment and data collection monitoring, productivity, and quality oversight. Study staff are responsible for recruitment, data collection, and implementation of study procedures. Statistical data and database team are responsible for REDCap database technical assistance, database management, data quality monitoring, and quantitative data analyses. Qualitative data and database team are responsible for qualitative database technical assistance, qualitative database management, and qualitative data analyses.

#### Composition of the data monitoring committee, its role and reporting structure {21a}

The National Institute on Aging (NIA) monitors this trial and an NIA monthly report will be submitted on participant screening and enrollment to NIA CROMS. A Data Safety Monitoring Board (DSMB) was created and is responsible for monitoring this trial. The DSMB acts in an advisory capacity to monitor participant safety, evaluate the progress of the study, and review procedures for maintaining the confidentiality of data, quality of data collection, management, and analyses. The responsibilities of the DSMB are fully outlined in the DSMB Charter, available in Additional file 3. The PI is responsible for responding to all recommendations of the DSMB. The individuals serving on the DSMB are independent of all study activities. DSMB members have no direct involvement with the study investigators or intervention. The DSMB members sign a Conflict of Interest Statement which includes current affiliations with pharmaceutical and biotechnology companies (e.g., stockholder, consultant), and any other relationship that could be perceived as a conflict of interest related to the study. Any questions regarding members’ independence to the study are addressed and corrected at that time. Reports are submitted annually to the DSMB. The report includes study status, participant descriptive information, and safety information including any AE/SAEs and study quality related to recruitment, retention, and completeness of study activities. Reports are modelled using NIA’s DSMB Reports Templates.

#### Adverse event reporting and harms {22}

Because the ENACT study will enroll participants who are age 70 and older and often have chronic medical conditions including cognitive impairment and/or functional limitations, adverse events (AEs) may occur. Examples of AEs include distress due to discussion of future medical care planning, interpersonal conflict related to discussions with others (including health care practitioners), and concerns related to how advance care planning is documented and communicated in the medical record. Although unlikely to be a consequence of the ENACT Group Visits, COVID-19 diagnoses and death will be assessed and reported as Adverse and Serious Adverse Events (SAE).

The PI is informed of SAE as soon as they occur and notifies the DSMB, NIA and COMIRB within 48 hours of knowledge of event. A report summarizing all AEs is sent to the NIA Program Officer and to the DSMB once a year, unless otherwise requested by the NIA staff or DSMB. Reporting occurs more frequently if there are SAEs. Expected SAEs are listed in the data and safety monitoring plan. Unanticipated Problems will be reported, including a corrective plan and measures to prevent reoccurrence, within 48 hours to COMIRB and NIA. COMIRB is required to report all AEs to the DHHS Office for Human Research Protection (OHRP), University officials, and sponsors.

We will classify AE as to severity, expectedness, and possible relatedness, based on NIA Adverse Event and Serious Adverse Event Guidelines. All events (possible AEs and unanticipated problems) will be evaluated by the PI to determine severity (mild, moderate, severe), expectedness (unexpected, expected) and relatedness (definitely, possibly, or not related to the intervention).

##### Severity

Most possible AEs that participants may experience will be mild, such as psychological distress due to discussion of future medical care planning or interpersonal conflict related to discussions with others, including health care practitioners. Moderate or severe AEs could be demonstrated by highly negative psychological distress reactions such as anger, uncontrolled crying, or early termination of an interaction with intervention. Per NIA policy, severity is not synonymous with seriousness.

- *Mild:* Events include awareness of signs or symptoms that are easily tolerated and minor causing no loss of time from normal activities. Symptoms do not require therapy or a medical evaluation; signs and symptoms are transient.
  - *Moderate:* Events introduce a low level of inconvenience or concern to the participant and may interfere with daily activities. They usually improve with simple interventions (e.g., a follow-up conversation, empathic statements, talking with family).
  - *Severe:* Events interrupt the participant’s normal daily activities and generally require treatment because otherwise they are usually incapacitating.

No hospitalizations or deaths related to the study interventions are expected. All deaths will be reported as soon as study staff are aware and within 24 hours of study PI’s knowledge of death. The report of death will be submitted to NIA Program Officer and DSMB Chair.

##### Expectedness

The majority of possible AE will be anticipated.

- *Unexpected* – Unexpected Serious Adverse Events (SAE) are those events that are not listed as expected in nature or severity to the known possible adverse impact of advance care planning counseling and have not been previously reported for ENACT Group Visits intervention. If such unexpected SAEs occur, they will be reported to the NIA Program Officer and to the DSMB Chair within 48 hours of study PI’s knowledge of SAE.
- *Expected*– Anticipated events are AE that are known to be associated with advance care planning counseling and related to the study interventions. This includes distress due to discussion of future medical care planning, interpersonal conflict related to discussions with others, including health care practitioners.

##### Relatedness

The possible AE/SAE relationship to the study intervention and/or participation is assessed by the site investigator and categorized as follows:

- *Definitely Related*: The event is clearly related to the advance care planning intervention – i.e. an event that follows a reasonable temporal sequence from study procedures.
- *Possibly Related:*The event follows a reasonable temporal sequence from study procedures or follows a known or expected response pattern to the suspected intervention but could readily have been produced by a number of other factors.
- *Not Related:*The event is clearly not related to the study intervention - i.e. another cause of the event is most plausible; and/or a clinically plausible temporal sequence is inconsistent with the onset of the event and the study intervention and/or a causal relationship is considered biologically implausible.

#### Frequency and plans for auditing trial conduct {23}

At the direction of the DSMB and the sponsor, there are no plans for an external, independent audit.

#### Plans for communicating important protocol amendments to relevant parties (e.g. trial participants, ethical committees) {25}

All protocol modifications will be communicated to the NIA at the time of approval by the IRB and will be summarized with each report to the DSMB and NIA. Communication with study investigators is monthly at all-team meetings. Changes will be communicated to trial participants when necessary and as directed by the healthcare system, IRB, NIA and/or DSMB.

#### Dissemination plans {31a}

ENACT study findings will be broadly disseminated through presentations at academic conferences, publication in peer-reviewed academic journals, and widely through mechanisms designed to reach the lay public. All participants will be asked if they would like to receive study results when available. Formats for sharing out results will include newsletters, emails with brief videos, and invitations to in-person events where results will be shared. The study is registered with ClinicalTrials.gov. Research consent forms will include a statement directing participants to ClinicalTrials.gov for trial information, including submitted results from findings. Dissemination of results may also include 1) sharing the most recent version of the ENACT Group Visits Implementation Manual upon request; 2) seeking opportunities to present results to clinical primary care teams and settings that are non-academic (e.g., primary care value-based payment programs); 3) leveraging social media for broader and more diverse reach.

## Discussion

The ENACT study is an NIH Stage III efficacy study designed to test the efficacy of the ENACT Group Visits intervention in primary care settings. By training clinic-based facilitators and embedding the intervention within standard workflows, the study aims to test a scalable, billable, and person-centered ACP approach tailored for older adults, including individuals with possible MCI. This trial includes 480 diverse primary care patients, including Spanish-speaking participants and care partners, and is being implemented across eight clinics in two health systems. A notable strength is its multi-level, mixed-methods evaluation of implementation outcomes such as feasibility, acceptability, fidelity, and sustainability, with particular attention to adaptations for Hispanic/Latino populations. These features increase the real-world relevance and potential scalability of the intervention. This discussion highlights additional practical, operational and other relevant aspects of the clinical trial that have not already been covered as part of the SPIRIT guidelines.

The proposed trial outlines the components of the ENACT Group Visits intervention and planned implementation strategies to conduct the trial in 8 primary care clinics. Table 3 describes the core functions of this ACP group visit model, and the forms or structures that will be used in the ENACT Group Visits Intervention. The goal of defining the intervention using the function-form approach is to support measurement of intervention fidelity in different clinic settings and to catalog different forms that clinics may choose to implement the intervention in their clinic (32). The main implementation strategies are facilitator and clinic staff training through clinic meetings and online modules for Group Visit facilitators; quarterly learning collaboratives for facilitators; and technical assistance to facilitators and clinic staff by the study team and PI.

The ENACT study design incorporates some pragmatic features, as outlined in Table 6, using the PRECIS-2 framework (54). Some of the more pragmatic features include patient inclusion criteria, primary outcome selection, and flexibility of the ENACT Group Visit intervention. For example, the inclusion of patients with possible cognitive impairment is more generalizable to primary care settings because requiring a formal diagnosis of MCI or early ADRD to be included in the intervention would be less practical and generalizable for most clinics. The informed consent process focuses on assessing the participant’s capacity to demonstrate ability to understand the research study, which is like a clinician’s ascertainment of ability to understand ACP during a clinic visit. The primary outcome of an ACP document in the EHR is pragmatic because it can be assessed without needing patient-report and EHR-based ACP documents are a common way that primary care practices and health systems measure the ACP process. Finally, the design of the ACP group medical visit is pragmatic because it has core functions that can be flexibly adapted by group visit facilitators and clinic staff to fit the needs, preferences, and resources of the local clinic.

### Inclusion of Spanish-speaking older adults

To provide an opportunity for older patients who speak Spanish to participate in ENACT Group Visits, we will adapt the protocol to allow for study participation entirely in Spanish. One of the two health systems includes a network of Federally Qualified Health Centers that serves a large population of Spanish-speaking patients. Cultural adaptation will include a team-based approach for translating all participant materials as well as facilitator guides into Spanish in collaboration with a certified Spanish translator. Study implementation includes employing bilingual, bicultural research team members to recruit and enroll patients, as well as bilingual facilitators to be able to offer the ENACT Group Visits intervention entirely in Spanish. Because all data collection will include patients and care partners who speak Spanish, this study will assess the ACP outcomes and implementation outcomes (i.e., acceptability, feasibility, adaptation) of the intervention among this population.

### COVID-19 pandemic considerations

The global COVID-19 pandemic conveyed greater-than average risk specifically for this study’s target population of older adults, that required specific planning, systems for ongoing monitoring, contingency planning, and consideration of adaptations. Multiple safety accommodations were planned for use during the duration of the U.S. COVID-19 public health emergency. Specifically, county-level COVID-19 infection rates, as indicated from the Centers for Disease Control (CDC) website, were monitored and used to inform the level of precautions for the ENACT study. For example, when county-level infection rates were deemed high, any planned ENACT Group Visits interventions would be held virtually. For medium levels, masks were required by participants and staff and each participants’ status of symptoms was checked 2 days prior to attending a Group Visit and 2 weeks following the group. For low levels, groups proceeded as planned. After May 11, 2023, when COVID-19 community levels and COVID-19 community transmission levels were no longer calculated through the CDC, the study protocol shifted to adhere to safety precautions required by participating clinics/health system.

## Conclusion

The ENACT study is a large, multi-site, randomized controlled trial to test the efficacy of an ACP group medical visit compared to mailed ACP materials among older patients in diverse primary care clinics. The Engaging in Advance Care Planning Talks (ENACT) Group Visits intervention will be implemented for English or Spanish-speaking populations and enables inclusion of older adults with possible cognitive impairment and their family care partners. Study findings will enable primary care clinicians, staff, and leaders to make informed decisions about implementing a potentially sustainable, group-based ACP model.

## Figures and Tables

**Table 1.** Overview of ENACT Group Visits intervention format and description

Group Visit format	Description
Group visit agenda and timing	Check-in process (30 min) Introductions and rapport building (20 min) ACP discussion using facilitation skills, a facilitation guide, and ACP resources (60 min) Individual Goal setting (10 min) Optional: Group workshop time to complete advance directives
Group A content	Introduction: Share reasons for coming to talk about ACP Share personal ACP stories and experiences Consider personal values Discuss role of surrogate decision makers, including choosing and sharing wishes with decision makers Ask questions related to ACP Discuss ACP educational resources and advance directives Set goals for personal ACP actions
Group B content	Re-connection: Review individual ACP goals Discuss flexibility for surrogates in decision making Consider future health care choices, based on questions or concerns Plan for conversations with health care professionals Ask questions related to ACP Discuss aspects of advance directives, such as having them available and accessible Opportunity to update ACP preferences (i.e., healthcare decision maker, future medical preferences in the medical record) via group visit facilitator Set next goals for personal ACP actions

**Table 2.** Inclusion and exclusion criteria by participant types in the ENACT study.

Participant Type	Inclusion Criteria	Exclusion Criteria
Patient	Age ≥ 70 years At least one primary care visit in the past year No recent ACP document (within the past 2 years) in the EHR Preferred language is English or Spanish	Unable to demonstrate capacity to understand the study Lack access to a telephone for follow-up calls Unable to travel to clinic Moving out of the area within 6-months Unable to participate in group visits due to hearing impairment as determined by clinic and/or study staff Household member with the same address as the patient participant who is already enrolled Lack of insurance coverage for clinic visits
Care Partners of patients with possible cognitive impairment	Age ≥ 18 years Preferred language is English or Spanish Care partner to a consented patient participant who has screened positive for possible cognitive impairment	Unable to demonstrate capacity to understand the study Lack access to a telephone for follow-up calls Unable to travel to clinic Moving out of the area within 6-months Unable to participate in group visits due to hearing impairment as determined by clinic and/or study staff
Clinic Staff	Multidisciplinary team member at a participating primary care clinic Preferred language is English	None

**Table 3.** ENACT Group Visits Intervention Core Functions, Associated Structures and Rationale.

Core Functions	Intervention Structures to Achieve Core Functions	Rationale
Peer and expert-based learning	Multiple patients participate in a group medical visit Participants are 8-10 patients, who have the option to bring a care partner* Clinic infrastructure includes: Adequate seating; Video capability; White erase board; Water	Based on prior data, more diverse perspectives were shared when there were 8-10 patients As few as 6 patients can support a group dynamic, though the range of experiences is smaller (17)
Rapport building to support sharing about ACP	Introductions and rapport building segment (20 min) at each group visit session	Facilitate openness, trust, and meaningful dialogue among patients and facilitators
ACP Education	Facilitator-guided discussion, covering personal values, surrogate decision-maker roles, future healthcare choices, flexibility in decision-making, and advance directives	Engagement Theory (patient-level) (28,29), Collaborative Learning Theory (interpersonal level) (24), and strengths of group visits (clinic-level) (13)
Evidence-based ACP resources	PREPARE^™^ Colorado Advance Healthcare Directive form and instructional pamphlet Educational ACP videos	ACP resources are designed to accommodate low health literacy, support informed ACP decision-making, and be accessible to diverse populations
ACP discussions from different healthcare perspectives	Two multidisciplinary facilitators - Represent two different disciplines (i.e., MD or APP, social worker; care manager)	Supports varied learning styles and healthcare perspectives to facilitate a group and engage patients in behavior change related to ACP
Individualized ACP goal setting	Visits are 1 month apart Include use of an ACP goal-setting worksheet	Two visits support goal setting and follow up. Goal-setting worksheet used in the preliminary studies, and adapted from a Veterans Health Administration-based ACP group visit model (33)
Documentation of patient’s ACP preferences and/or process	Facilitators update patient preferences (e.g., healthcare decision-maker, ACP documentation, any code status decision) directly into the electronic health record (EHR) Visit note sent to primary care clinicians to share ACP preferences	Clinician documentation of ACP supports accuracy, accessibility, and awareness for other healthcare teams and patients
Reimbursable ACP process in a clinic setting	At least one facilitator bills for the visit (i.e., MD or APP) Document as Evaluation and Management visits	Supports financial sustainability and scalability of ACP group visits in primary care through existing reimbursement frameworks

* *Care partner* = A trusted person, such as a friend, partner, spouse, or family member, who was identified by the patient participant as someone they would want to be part of ACP; *APP =* advanced practice provider

**Table 4.** ENACT study measures with time points and analytic plan.

Domain and Key Measures		Assessment Time		Analytic Plan
		Baseline	6-months	
**New ACP Documentation**	ACP documents in EHR	X	X	1° Outcome
**ACP Readiness**	4-item ACP Engagement Survey (34)	X	X	2° Outcome
**Decision Self-Efficacy**	Decision Self-Efficacy Scale (35)	X	X	2° Outcome
**Quality of Communication**	Quality of Communication General Communication Skills Scale (36,37)	X	X	2° Outcome
**ACP Composite**	ACP documents in EHR and clinician documentation		X	2° Outcome
**Cognitive Measures**				
**Objective Cognition**	Telephone Montreal Cognitive (T-MoCA) Assessment (24, 27)	X		Stratify, covariate
**Subjective Cognitive Decline**	3-items adapted from Subjective Cognitive Decline measures (38,39)	X		Covariate
**Other Covariates**				
**Demographics (Patient and Care Partner)**	Age, gender, preferred language, race, ethnicity, marital status, years of education,	X		Covariate
**Self-rated Health**	PROMIS Global Health (40)	X	X	Covariate
**Medical Conditions**	EHR: Charlson Comorbidity Score, body mass index, diabetes, hypertension, depression, anxiety, HDL level, dementia	X		Covariate
**Prior ACP Experience**	5-items (e.g., “*Have you ever had to make life-threatening medical decisions?*”) (26,31)	X		Covariate
**ACP Communication**	3-items (e.g., “*If you become too sick to make your own decisions, can you think of ANYONE in your life right now, such as family or friends, who MAY be able to help make medical decisions for you?*”)	X		
**Social Factors**	Medical Outcomes Study (MOS) Social	X		Covariate
	Support Survey(41)			
	Two-items from PRAPARE(42)	X		Covariate
**Other Measures**				
**Intervention Fidelity**	Fidelity Checklists of Group Visit recordings	Qualitative descriptive analysis		
**Implementation Outcomes (i.e., Acceptability, Feasibility)**	Interviews/focus groups; Acceptability of Intervention; Intervention Appropriateness Measure; Feasibility of Intervention (36)		X	Mixed Methods

**Table 5.** ENACT study qualitative data collection with time points and analytic plan

Data collection method	Participants	Purpose	Timing and Frequency	Analysis
Video recordings of group visits	All group visit attendees (patients, care partners, facilitators, study team members)	To assess intervention fidelity (i.e., adherence to group visit intervention protocol) across all group visits and compare to primary outcomes.	Recordings collected of each group visit across clinics	Rapid qualitative analysis
Periodic reflections	Group visit facilitators	To a) support ongoing reflection and problem-solving during implementation, and b) learn about factors that influenced implementation, (i.e., context, external environment, adaptations, and implementation strategies).	Approximately twice during group visit implementation	Rapid qualitative analysis
Learning Collaboratives	Group visit facilitators	To provide opportunities for facilitators across clinics to share knowledge during implementation, including asking questions about challenges and sharing experiences.	Twice per year (approximately 6-months apart)	Rapid qualitative analysis
Patient and Care Partner Interviews	Patients with possible MCI (T-MoCA score ≤ 17) and their care partners	To understand and assess the feasibility and acceptability of the group visit intervention for patients with possible MCI and their care partners.	6-months after group visit attendance (intervention arm) or mailing of ACP materials (control arm)	Content analysis
Post-intervention leadership interviews	Clinic leaders (medical directors) and administrators (practice administrator)	To assess feasibility and acceptability of ENACT at the clinic level, including whether ENACT aligned with or supported clinics' goals, necessary resources, and perceived patient impact,	Within 3 months of finishing patient enrollment into the study at the respective clinic	Rapid qualitative analysis
Post-intervention staff focus groups	Clinic staff involved in group visit implementation (facilitators, nurses, medical assistants, schedulers)	To assess feasibility and acceptability of ENACT for clinic staff involved in implementation, including logistical considerations, resources, and experiences.	Within 3 months of finishing patient enrollment into the study at the respective clinic	Rapid qualitative analysis
Sustainment interviews	Group visit facilitators	To understand whether clinics have sustained group visits, including factors that influenced clinics' decisions to either continue or stop offering ACP group visits.	6-months post-intervention leadership interview/ focus group, or 8-12 months after the last group visit conducted at the clinic	Rapid qualitative analysis

**Table 6.** Rating of Pragmatic Features of the ENACT Study.

PRECIS-2 Domain	ENACT Study Design	Pragmatic-Explanatory Rating
Eligibility Criteria	Age 70 or older, receiving primary care at a study clinic, no recent ACP documents in EHR in the past 2 years, ability to participate in English or Spanish, and ability to demonstrate study consent	3 – Allows individuals with possible cognitive impairment to participate if they can demonstrate informed consent; Allows English or Spanish speaking patients (but not other preferred languages)
Recruitment	Patient-level recruitment by study staff (rather than clinic teams)	1 – Tailored recruitment using multiple modalities, including in-person at primary care clinics
Setting	Eight primary care clinics in two health systems	3 – Clinics serve diverse patient populations; one is an academic health system, one is a federally-qualified health system
Organization	ENACT Group Visits (intervention arm) are a complex intervention, supported by 1-2 study staff. Mailed ACP materials (control arm) are low cost to print and mail	2 – ENACT intervention involves specific training for facilitators, a clinic team-based approach, a clinic conference room, ACP resources
Flexibility: delivery	ENACT Group Visits have core components that are designed to be delivered with some flexibility	3 – Facilitators and clinic staff can adapt the intervention to the needs of their patients or clinic (i.e., facilitator roles; session guides; timing of visits)
Flexibility: adherence	Study activities include learning collaboratives and facilitator summaries to provide input and feedback to Group Visit facilitators	3 – Study staff provide optional technical support and feedback to Group Visit facilitators. Study staff will identify if a group visit is “highly adapted” but will not intervene or remove from analysis
Follow-up	6-month follow up in the EHR and phone-based survey/interview	4 – Primary outcome does not require patient-level follow up. There is only one follow up time point, conducted by phone
Primary outcome – relevance to participants	Primary outcome is ACP documentation in the EHR	4 – Primary outcome is relevant to patients, clinicians, health systems, and payors (even though it is not the only relevant ACP outcome)
Primary analysis	Analysis will be intention-to-treat	5 – All data will be included, even if participants do not participate in their assigned study arm or if the group visits are “highly adapted”

## Abbreviations

ACP: Advance Care Planning
AE: Adverse Event
APP: Advanced Practice Provider
ADRD: Alzheimer’s Disease and Related Dementia
CDC: Centers For Disease Control
COMIRB: Colorado Multiple Institutional Review Board
CTSI: Clinical and Translational Science Institute
DSMB: Data Safety Monitoring Board
DISC: Development and Informatics Service Center
HER: Electronic Health Records
ENACT: ENgaging in Advance Care planning Talks Group Visits intervention
FAQ: Frequently Asked Questions
IRB: Ethics Review Board
GLMM: Generalized Linear Mixed Model
ICU: Intensive Care Unit
ICC: Intra Class Correlation
MCI: Mild Cognitive Impairment
MoCA: Montreal Cognitive Assessment
MOS: Medical Outcomes Study
NIA: National Institute on Aging
OHRP: Office for Human Research Protection
POLST: Portable Orders for Life-Sustaining Treatment
PRECIS-2: Pragmatic-Explanatory Continuum Indicator Summary 2
PCPs: Primary Care Providers
PI: Principal Investigator
QOC: Quality of Communication
RCT: Randomized Controlled Trial
REDCap: Research Electronic Data Capture
RUCA: Rural-Urban Commuting Area Codes
SAE: Serious Adverse Event
T-MoCA: Telephone Montreal Cognitive Assessment
UCSF: University of California, San Francisco
CU-AMC: University of Colorado Anschutz Medical Campus
US: United States
